# Overview of nerve entrapment syndromes in the foot and ankle

**DOI:** 10.1007/s00264-025-06469-5

**Published:** 2025-03-05

**Authors:** Milos Bojovic, Sanja Dimitrijevic, Bruno C. R. Olory, Cristiano Eirale, Omar AlSeyrafi, Abdulrahman Abdulla AlBaker, Branislav Krivokapic, Danilo Jeremic, Pieter DHooghe

**Affiliations:** 1 Aspetar Orthopedic and Sports Medicine Hospital, Doha, Qatar; 2 Special Hospital For Cerebral Palsy And Developmental Neurology, Belgrade, Serbia; 3 Institute for Orthopaedic Surgery “Banjica”, Belgrade, Serbia; 4 Faculty of Medicine, University of Belgrade, Belgrade, Serbia

**Keywords:** Tunnel syndromes, Foot and ankle, Nerve entrapment, Diagnosis, Treatment, Nerve compression

## Abstract

**Purpose:**

Tunnel syndromes around the foot and ankle are underrecognized and frequently misdiagnosed nerve entrapments that can significantly impact patients' quality of life. This review aims to provide a comprehensive overview of the etiology, clinical presentation, diagnostic challenges, and management strategies for these syndromes, focusing on the sural nerve, deep peroneal nerve, tibial nerve, medial plantar nerve, and inferior calcaneal nerve.

**Methods:**

A thorough literature review was conducted, examining studies and case reports on nerve entrapments in the foot and ankle. The review covers the clinical assessment, differential diagnosis, and treatment options, including conservative and surgical interventions.

**Results:**

Tunnel syndromes of the foot and ankle can arise from various causes, including trauma, anatomical variations, repetitive strain, and systemic conditions. Clinical manifestations often include burning pain, tingling, and motor weakness, depending on the affected nerve. Accurate diagnosis relies on a detailed patient history, physical examination, and adjunctive tests such as electrodiagnostic and imaging. Conservative treatments, such as physical therapy, orthotics, and corticosteroid injections, are often effective, while surgical decompression is reserved for refractory cases.

**Conclusions:**

Recognizing and diagnosing tunnel syndromes in the foot and ankle is essential for effective management and preventing permanent nerve damage. A systematic approach that integrates clinical evaluation and appropriate imaging can improve patient outcomes. Timely intervention, whether conservative or surgical, is crucial for alleviating symptoms and restoring function.

## Background

Nerve entrapments in the foot and ankle are often overlooked, resulting in delayed treatment. Peripheral nerve entrapment, known as "tunnel" syndrome, occurs when a nerve is compressed within anatomical structures like bones, muscles, or fibrous tissues. This highlights the need for better awareness and refined approaches to diagnosis and treatment, as these syndromes significantly affect patient care and quality of life.

Peripheral nerves transmit sensory and motor signals but can be injured or compressed, causing tunnel syndrome symptoms that depend on the affected nerve. This article will use "tunnel syndromes" for clarity. These syndromes arise from various factors, including trauma, metabolic disorders, infections, vascular issues, anatomical anomalies, and repetitive activities. [1].

A clinical evaluation is essential for patients with neurovascular symptoms in the foot and ankle. [2]. Differential diagnosis is vital since various conditions can produce similar symptoms, like sharp pain and abnormal tingling from nerve pressure or poor blood flow. [2]. Muscle weakness and deep pain may occur with motor nerve involvement, affecting muscle groups or joints locally and distally. [3]. Differentiating sensory and motor nerve involvement is crucial for accurate diagnosis. Electromyoneurography is useful, but its reliability varies with the examiner and assessed nerve. [4]. A comprehensive clinical history and physical examination are essential, supported by additional tests as needed. [4].

Management of tunnel syndromes targets nerve compression causes through conservative or surgical treatments. While splinting and rest can aid repetitive movements, they may not be enough for compression from fractures, soft tissue, or anatomical issues. Addressing systemic or hormonal factors is also important to alleviate nerve compression. [5]. Timely intervention is critical, as delays can increase the likelihood of irreversible nerve damage [6].

When conservative treatments are appropriate, options such as immobilization, rest, therapeutic exercises, heat application, and anti-inflammatory medications should be considered. Monitoring patient response is crucial. If symptoms persist or worsen, corticosteroid injections may be an option before surgery, providing relief within 48 h as inflammation decreases. [7]. However, repeated corticosteroid injections should be approached with caution due to their potential to weaken tendons and joints over time [8]. Healthcare providers and patients need clear treatment timelines to avoid delays that can impede nerve recovery. There's no universal guideline for injection frequency in lower limb nerve entrapments, but studies indicate that more than one therapeutic injection is generally not recommended due to tendon and bone risks.

Surgical intervention is considered when conservative treatments fail to provide adequate relief. Surgical options may include direct visualization and decompression of the affected nerve, as well as procedures such as neurectomy, neurolysis, tenosynovectomy, arthrodesis, or osteotomy to expand the space within the tunnel [9].

## Sural nerve entrapment

The sural nerve, a primarily sensory nerve, can become compressed at various points along its pathway, resulting in discomfort in the calf, lateral ankle, and foot. This condition is frequently observed in runners and individuals with a history of recurrent ankle sprains [3]. Compression or trauma to the sural nerve may lead to symptoms such as neuropathic pain, burning sensations, heightened skin sensitivity, and in some cases, symptoms resembling Complex Regional Pain Syndrome (CRPS). The sural nerve's anatomical path makes it prone to compression or traction injuries, descending along the posterior lower leg and behind the lateral malleolus. A common site of entrapment is where the nerve pierces the crural fascia, typically in the middle to lower third of the calf [10].

**Fig. 1:**
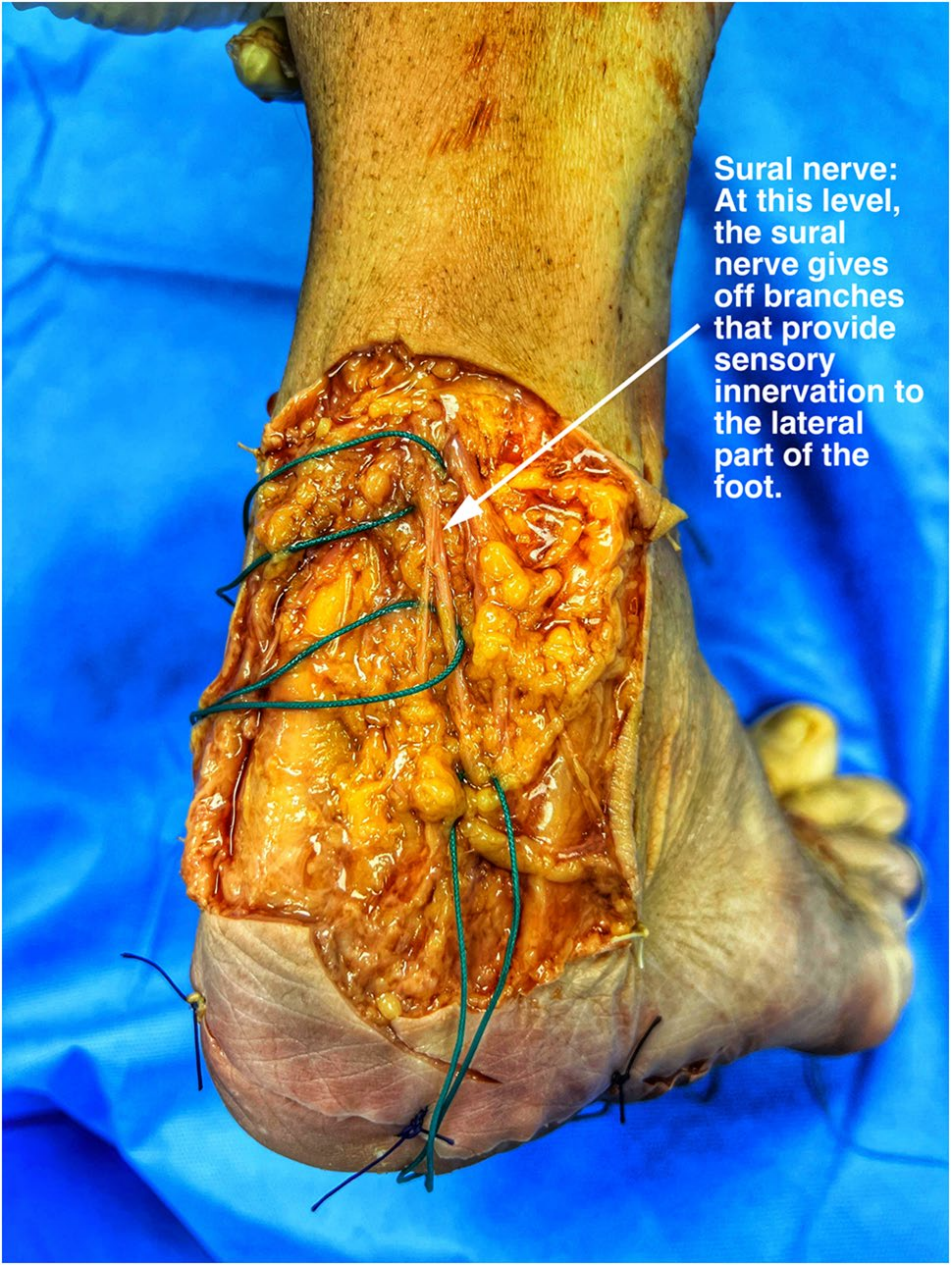
Sural nerve, cadaver dissection: The sural nerve travels within the subcutaneous tissue of the lower leg. It passes posterior to the lateral malleolus and enters the foot

The sural nerve has two main parts: the medial cutaneous sural nerve from the tibial nerve and the lateral sural cutaneous nerve branch from the common peroneal nerve. About 20% of people show variations in its formation or path.[11]. The nerve starts at the back of the knee and runs between the calf muscles. It travels alongside the small saphenous vein and next to the Achilles tendon, providing sensory innervation to the ankle, posterior calf, and lateral heel and foot. (Fig. 1) [10].

Sural nerve compression is often overlooked as a cause of exercise-induced leg pain, but it can arise from soft tissue growths, scarring, ganglia, surgeries, and vein inflammation. External factors like tight footwear, including ski boots and casts, can worsen the issue. The crural fascia protects but may also compress the nerve, limiting mobility. Repetitive activities, such as running, increase strain on the nerve, while repeated ankle sprains can lead to fibrosis and entrapment. In severe cases, fascia constriction may immobilize the nerve, increasing risk of traction injuries during intense foot movements. [6].

**Fig. 2:**
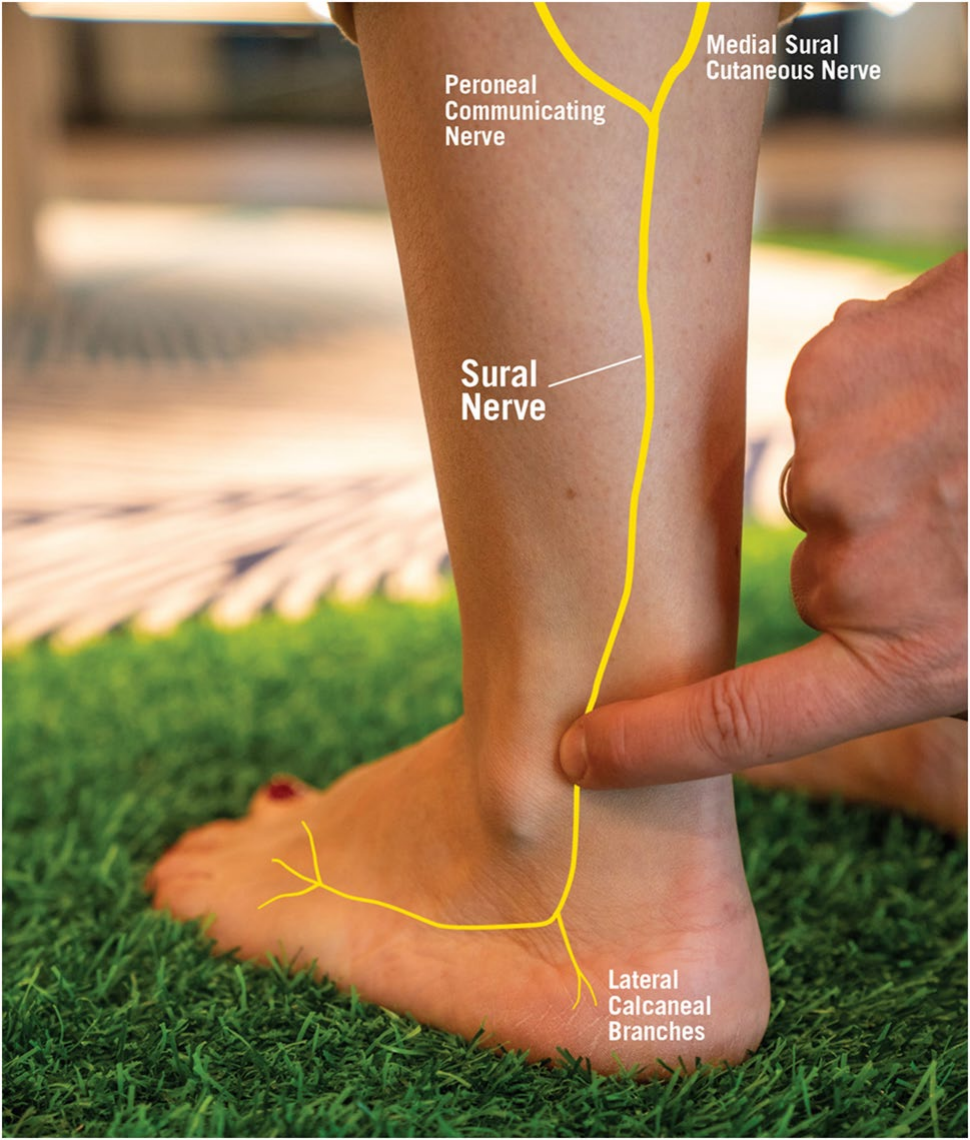
Point of maximal tenderness of the sural nerve: Branches of tibial nerve and common peroneal nerve creating the sural nerve On physical exam typical tenderness is behind the lateral malleolus, lateral to Achilles tendon. Reprinted with permission from the Aspetar journal

Patients often report sharp pain or unusual sensations localized to the sural nerve distribution. However, clear physical indicators may not always be present. Sural nerve compression is frequently misdiagnosed as an Achilles tendon issue, prompting athletes to seek treatment for what they assume is recurrent tendinopathy [12]. Upon clinical examination, tenderness and potential swelling may be identified in the calf or the area posterior and inferior to the lateral malleolus (Fig. 2).

Passive inversion of the ankle can reproduce sural nerve pain, triggered by plantar flexion and inversion.t [13]. Sensory disturbances like hypoaesthesia or hyperaesthesia often occur in the lateral foot and ankle. Tinel's test may not effectively diagnose sural nerve entrapment, but localized pressure can worsen symptoms, referred to as the Hoffman-Tinel’s sign. [10]. Electromyography (EMG) can provide additional diagnostic information, though abnormal findings are only observed in a subset of patients [14]. Differential diagnosis should include other potential causes of neuropathy, such as sciatic nerve entrapment.

## Treatment options for sural nerve entrapment

Initial treatment for sural nerve entrapment emphasizes conservative methods to alleviate irritation. Cold therapy and anti-inflammatory medications reduce inflammation. Removing external irritants, such as poor-fitting footwear, is crucial. Physical therapy may help mobilize the nerve and reduce compression from fibrous adhesions.

Ultrasound-guided corticosteroid or local anesthetic injections can be delivered at various sites along the nerve, typically 10–15 cm above the lateral malleolus or just behind it, where the gastrocnemius muscle meets the Achilles tendon. Treating the root cause of nerve compression is crucial for effective treatment, as eliminating the source often enables nerve regeneration and alleviates symptoms. [14].

If conservative measures fail, surgical options may be considered, including removing structures that compress nerves, like ganglia or Baker’s cysts. Neurolysis may be needed if a nerve is encased in scar tissue, and fasciotomy could alleviate excessive nerve tension during foot movements. Early identification and treatment of sural nerve entrapment can help patients regain daily function.

## Deep peroneal nerve (DPN) entrapment and anterior tarsal tunnel syndrome

The deep peroneal nerve (DPN), also referred to as the deep fibular nerve, is a significant branch of the common peroneal nerve. DPN injuries commonly affect athletes such as runners, football players, ballet dancers, and basketball players, complicating diagnosis and management. Recurrent ankle sprains significantly increase the risk of DPN entrapment by compressing nerves as they pass through the ligamentum cruciforme and inferior extensor retinaculum. Additionally, pressure from the extensor hallucis longus tendon can also cause entrapment. [15].

**Fig. 3:**
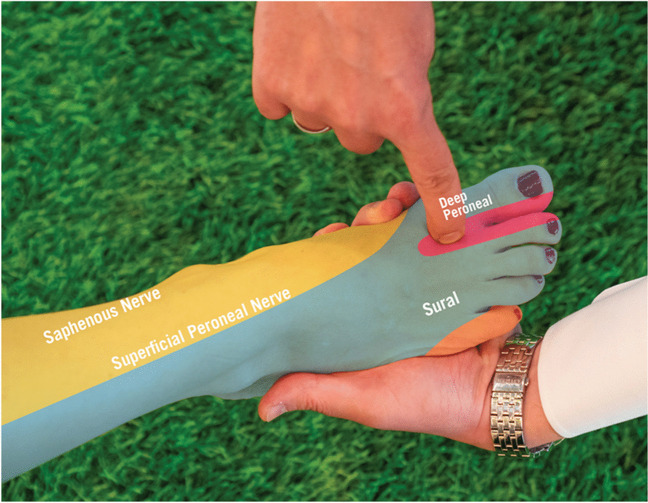
Interdigital web space with sensory innervation from the DPN: Area between first two toes where patients with entrapment often feels vague sensations. Reprinted with permission of Aspetar journal

The DPN is vital for motor function, innervating muscles like the tibialis anterior and extensor digitorum longus in the anterior lower leg. It also provides sensory innervation to the webspace between the first and second toes, coursing through the anterior tarsal tunnel above the talus. It splits into two branches, one for the extensor digitorum brevis and another supplying sensation to the metatarsophalangeal joints and the ankle joint, essential for foot stability before ending between the first and second toes. (Fig. 3) [16].

Tarsal tunnel narrowing and nerve compression can be caused by osteophytes, synovial cysts, ganglia, anomalous muscles, fractures, and inflammation. External factors such as high heels and trauma from repetitive stress or poor fitting shoes can aggravate the issue. Neuromas or aneurysms may also restrict tunnel space, with common triggers being foot trauma and extensor hallucis brevis muscle hypertrophy. [17], tight shoelaces, talonavicular bone spurs, ganglia, and high arches (pes cavus). DPN compression often arises from entrapment by the extensor hallucis longus tendon or inferior extensor retinaculum, exacerbated by prolonged plantarflexion, such as wearing high heels. Symptoms may worsen during sleep due to foot positioning, and rigid forefoot deformities may also play a role in developing the syndrome. [1, 10].

## Clinical presentation and diagnosis

Anterior tarsal tunnel syndrome manifests with both sensory and motor symptoms. Sensory issues may involve hyperaesthesia or hypoaesthesia between the first and second toes, along with tingling and tenderness over the deep peroneal nerve. Motor problems can hinder toe lifting, particularly the great toe, leading to atrophy of the extensor digitorum brevis. Patients often feel a burning sensation that intensifies with activity and can linger at rest, with nighttime pain worsening due to foot positioning stretching the nerve. Athletes, especially soccer players [18], are particularly prone to developing these symptoms due to repetitive trauma to the dorsum of the foot. Everyday activities, such as pressing on the nerve with objects like shoelaces or keys, may trigger discomfort.

Diagnosis of anterior tarsal tunnel syndrome typically includes clinical tests like Tinel’s sign, which usually shows positivity over the extensor hallucis brevis muscle. The "squeeze test," which compresses the metatarsal heads while applying pressure to the deep peroneal nerve, aids in confirming the diagnosis. Electrodiagnostic studies, help differentiate this condition from other neuropathies, such as peroneal nerve compression or L5 level lumbar radiculopathy.[18].

## Treatment approaches

Non-surgical management is the primary treatment for anterior tarsal tunnel syndrome, focusing on patient education, medications, local injections, physical therapy, and lifestyle adaptations. Conservative methods include rest, maintaining foot neutrality to reduce nerve compression, and anti-inflammatory drugs. Corticosteroid injections can address persistent pain. Physical therapy is crucial for athletes, improving proprioception and strengthening peroneal muscles. Medications such as anticonvulsants and tricyclic antidepressants aid in managing nerve-related inflammation and pain. If conservative treatments fail, surgical decompression may be recommended to relieve nerve entrapment and prevent neuroma or further damage. [15].

## Tibial nerve (TN) entrapment and tarsal tunnel syndrome (TTS)

The tibial nerve (TN) is susceptible to entrapment at specific sites as it approaches the ankle, with one of the most recognized locations being the tarsal tunnel (TT), also referred to as the tibiotalocalcaneal or Richet's tunnel. This anatomical structure is situated beneath the flexor retinaculum in the medial aspect of the ankle. Compression of the TN or its branches within this tunnel results in a condition known as tarsal tunnel syndrome (TTS). Although TTS is less prevalent compared to other nerve compression syndromes, it is often overlooked and may be incorrectly diagnosed as plantar fasciitis [19].

**Fig. 4:**
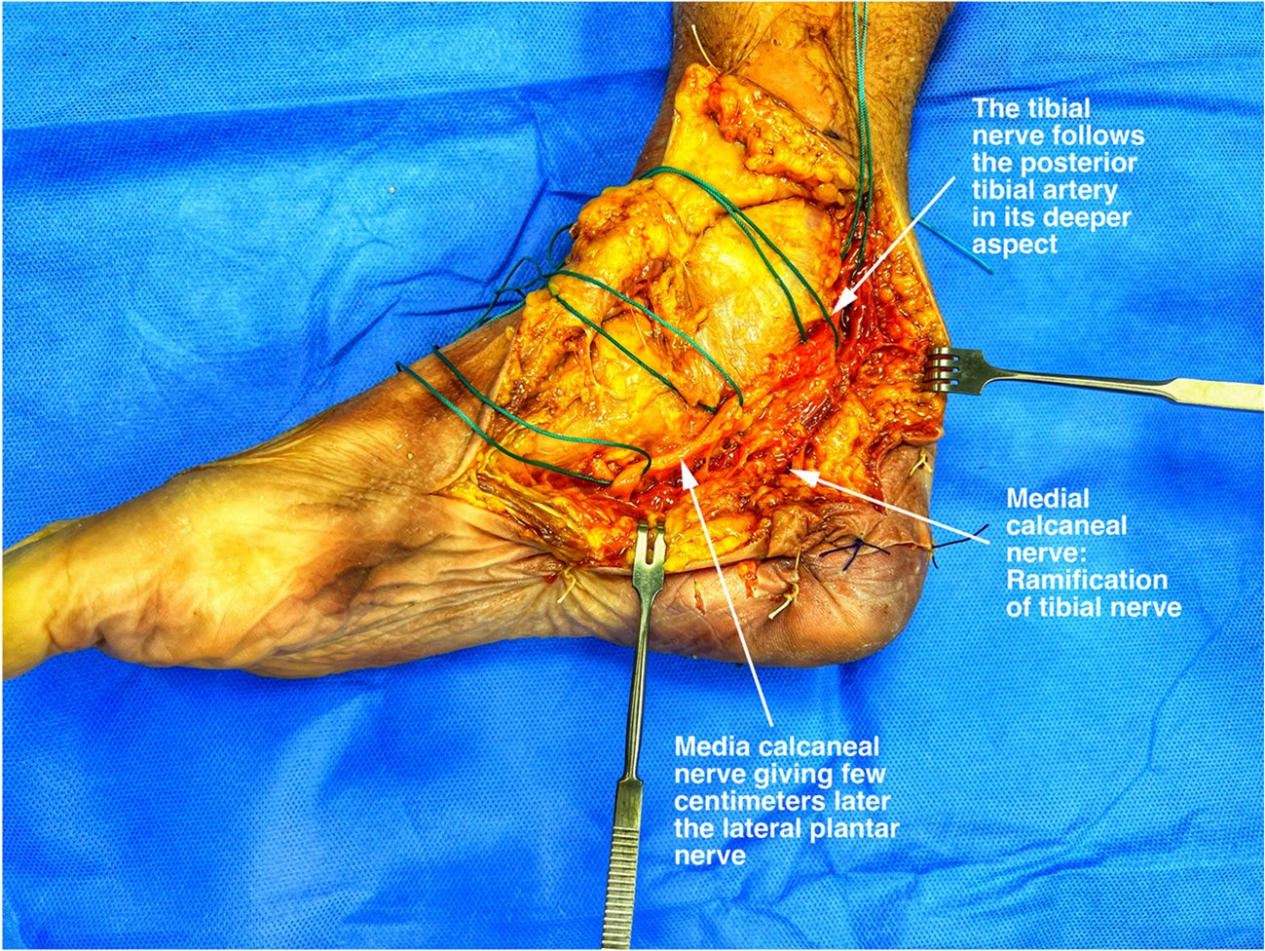
Tibial nerve, cadaver dissection: Tibial nerve lies posterior to its accompanying blood vessels. Medial calcaneal nerve arises from the tibial nerve

The tarsal tunnel is an important channel for nerves, blood vessels, and tendons entering the foot. It is surrounded by the medial malleolus, calcaneus, flexor retinaculum, and soft tissues. Inside, the tibial nerve and the tendons of tibialis posterior, flexor digitorum longus, and flexor hallucis longus run together, shielded by sheaths. (Fig. 4). The tibial nerve originates from the sciatic nerve in the upper leg and runs down the posterior leg to the medial ankle, where it passes through the tarsal tunnel, making it vulnerable to compression injuries. [20]. The tunnel's floor contains the talus, calcaneus, and distal tibia, with the flexor retinaculum above. The TN typically splits into the medial and lateral plantar nerves on entering the foot.

The TN controls lower leg muscles such as the popliteus, soleus, gastrocnemius, plantaris, tibialis posterior, flexor digitorum longus, and flexor hallucis longus. The lateral plantar nerve innervates the quadratus plantae, abductor digiti minimi, adductor hallucis, flexor digiti minimi brevis, interosseous muscles, and second to fourth lumbricals. The medial plantar nerve supplies the flexor digitorum brevis, abductor hallucis, flexor hallucis brevis, and first two lumbricals, along with sensory input for the outer sole and fifth toe.[10].

## Causes and risk factors

Tibial nerve compression can arise from a range of internal and external factors [21] External factors contributing to TTS include trauma, foot deformities, obesity, and systemic diseases like rheumatoid arthritis and diabetes. Internal causes involve fibrotic changes, tendon inflammation, bone spurs, and tumours. TTS is linked to conditions like tarsal coalitions and tight footwear, especially in athletes, particularly runners with excessive foot pronation.[22]. Traumatic injuries and associated bleeding or scar formation in the posterior ankle region may also precipitate TTS.

## Clinical presentation

**Fig. 5:**
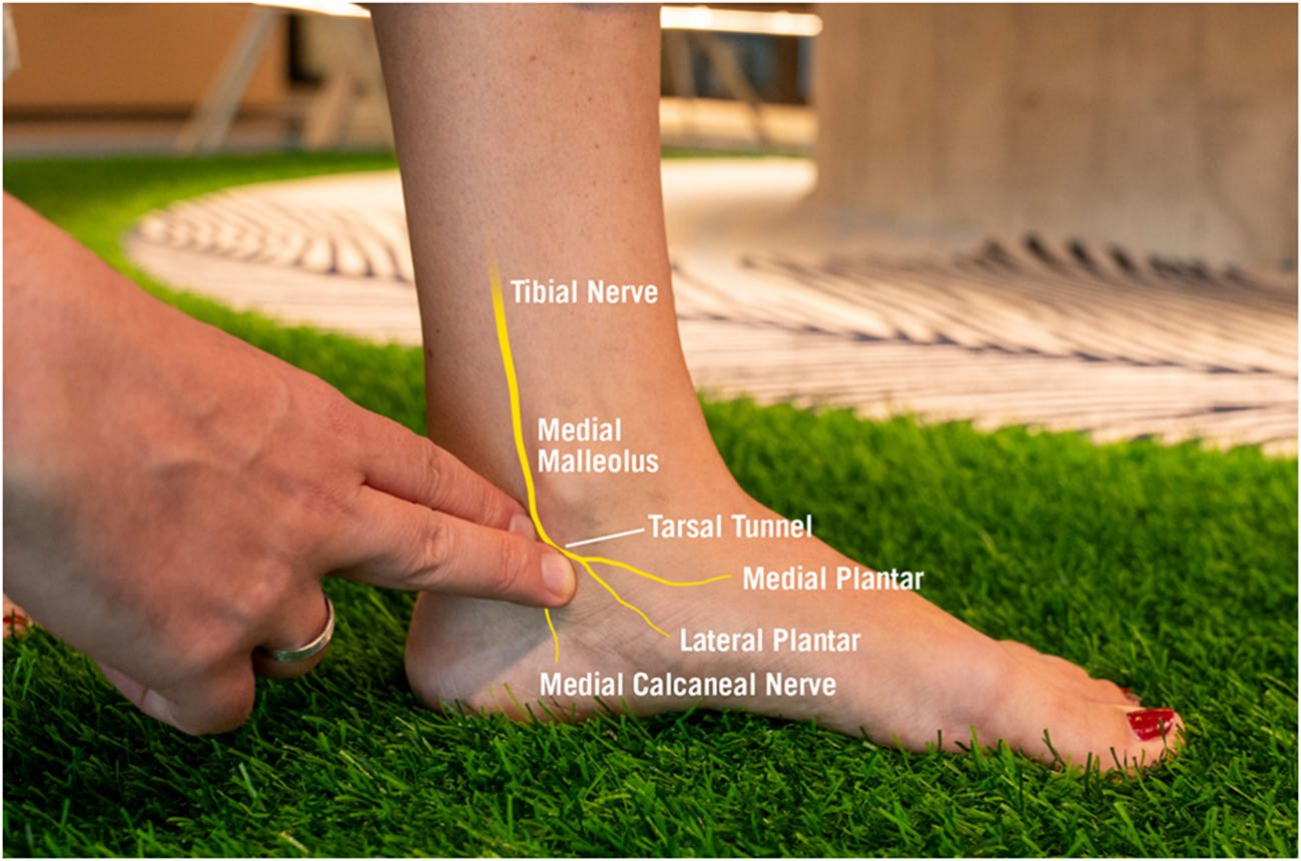
Palpation of the tibial nerve in the tarsal tunnel: There can be local tenderness at the medial ankle over the tarsal tunnel. A Tinel’s sign may be elicited with the fingers. Reprinted with permission of the Aspetar Journal

Patients with TTS often feel pain and numbness in the medial plantar nerve area, impacting the foot's inner side and the first three toes. Symptoms like burning or tingling may intensify at night or after prolonged standing. Pain can radiate, sometimes mimicking sciatica. Objective detection is challenging, but diminished sensation and reduced two-point discrimination suggest nerve compression. A positive Tinel’s sign is often seen, and decreased sweating in the affected region may also occur. (Fig. 5). Swelling around the medial ankle may be observed during examination, and maneuvers like foot eversion, dorsiflexion, and toe abduction can trigger pain and paresthesia in the medial plantar nerve area. Motor deficits might not be immediately noticeable due to muscle compensation. [23].

## Diagnostic evaluation

The triple compression stress test (TCST), also referred to as the dorsiflexion/eversion test, is a valuable diagnostic tool for TTS. This test involves dorsiflexing the ankle, everting the foot, and applying pressure to the tibial nerve, with symptom reproduction indicating a positive result. Studies have demonstrated that the TCST has a sensitivity of 85.9% and a specificity of 100%, making it a reliable diagnostic method [24]. Additional diagnostic modalities, including X-rays, ultrasound, CT scans, MRI, and electromyography (EMG), may be employed to confirm the diagnosis and differentiate TTS from other conditions such as plantar fasciitis, Morton's neuroma, and lumbar radiculopathy [25–28].

## Treatment approaches

Non-surgical treatment effectively manages TTS by reducing pain, inflammation, and nerve stress. If nerve conduction is impaired, conservative methods may fail. Strategies include modifying activity, using pain relief medications like acetaminophen and NSAIDs, and neuropathic pain treatment with gabapentin or tricyclic antidepressants. Physical therapy methods such as cryotherapy, orthotic inserts, and stretching exercises aid symptom reduction. Strengthening exercises, kinesiology taping, and night splints may also help, along with ultrasound-guided aspiration of cysts and corticosteroid injections for relief. [2, 5].

Surgical intervention is considered when conservative measures fail, and a clear anatomical cause of compression is identified. The procedure typically involves releasing the flexor retinaculum from its attachment near the medial malleolus down to the sustentaculum tali. Additional decompression of the deep fascia of the abductor hallucis muscle may be necessary to relieve pressure on the nerve's distal branches [5]. Surgical outcomes are generally favourable when performed in well-selected cases with clear indications.

## Medial plantar nerve (MPN) entrapment

Medial plantar nerve (MPN) compression commonly occurs near the navicular tuberosity, where the nerve traverses a tunnel formed by bone, soft tissue, and muscle, specifically between the navicular bone and the abductor hallucis muscle. This condition is common in middle-aged runners, who often report aching or sharp pain in the medial arch of the foot while running.[22].

The MPN, a crucial branch of the tibial nerve, arises beneath the flexor retinaculum, running alongside the posterior tibial artery. It travels near the quadratus plantae and abductor hallucis before reaching the master knot of Henry. [29]. As it travels further, it follows the flexor digitorum brevis muscle's inner edge and divides into articular, muscular, and cutaneous branches. The cutaneous branches innervate the skin of the anterior sole, including the medial three and a half toes. The muscular branches control the abductor hallucis, flexor hallucis brevis, flexor digitorum brevis, and the first lumbrical muscles, while the articular branches serve the tarsal and metatarsal joints.

Running with the foot in a valgus position for long durations strains the MPN, risking entrapment behind the navicular tuberosity. Repetitive foot eversion may stretch the nerve in a tunnel formed by the abductor hallucis and the navicular bone. [30]. Persistent strain can lead to inflammation and compression. To reduce worsening symptoms, it's advised to avoid rigid arch supports in running shoes. Distinguishing MPN entrapment from tendon pathologies is challenging due to the closeness of flexor digitorum longus and flexor hallucis longus muscles.

## Clinical presentation and diagnosis

Patients with MPN entrapment often describe burning pain, tingling, and aching sensations localized to the inner arch of the foot. Symptoms worsen with physical activity, radiating to the first and second toes or heel. Patients often report tenderness behind the navicular tuberosity along the medial arch and may feel reduced sensation in the sole near the great toe. [30]. Excessive foot pronation, uneven surfaces, and narrow shoes can worsen symptoms. Previous foot injuries, particularly to the flexor hallucis longus tendon, are common. Gait analysis may reveal foot supination, relieving pressure on the medial arch. A physical exam, including the tibial nerve, is essential. In MPN entrapment, tenderness is noted over the medial arch, from the abductor hallucis muscle to the navicular tuberosity. The Tinel's test, which involves localized pressure, is often positive, aiding diagnosis. [10].

## Management strategies

Conservative treatment eases symptoms and tackles causes. Key steps involve discontinuing high-arched orthotics, modifying footwear for support, and adjusting training to prevent uneven surfaces and excessive pronation. Local injections or pulsed radiofrequency therapy can reduce inflammation and nerve irritation.[31]. Rest, shorter running distances, and anti-inflammatory medications are recommended for recovery. If ineffective, electromyography (EMG) assesses nerve dysfunction.

Surgical intervention is considered when non-surgical treatments fail to relieve symptoms. The procedure focuses on decompressing the nerve by releasing the fascia at the tender area. Careful assessment and selection of candidates for surgery are essential for favourable outcomes.

## Inferior calcaneal nerve (ICN) entrapment

Medial heel pain may stem from entrapment of the inferior calcaneal nerve (ICN), often resembling or accompanying plantar fasciitis. [32], a condition now recognized as the result of repetitive microtrauma to the plantar fascia, leading to degeneration and persistent inflammation. Studies conducted in the late 1980s by Baxter suggest that ICN entrapment may account for up to 20% of heel pain cases [33].

**Fig. 6:**
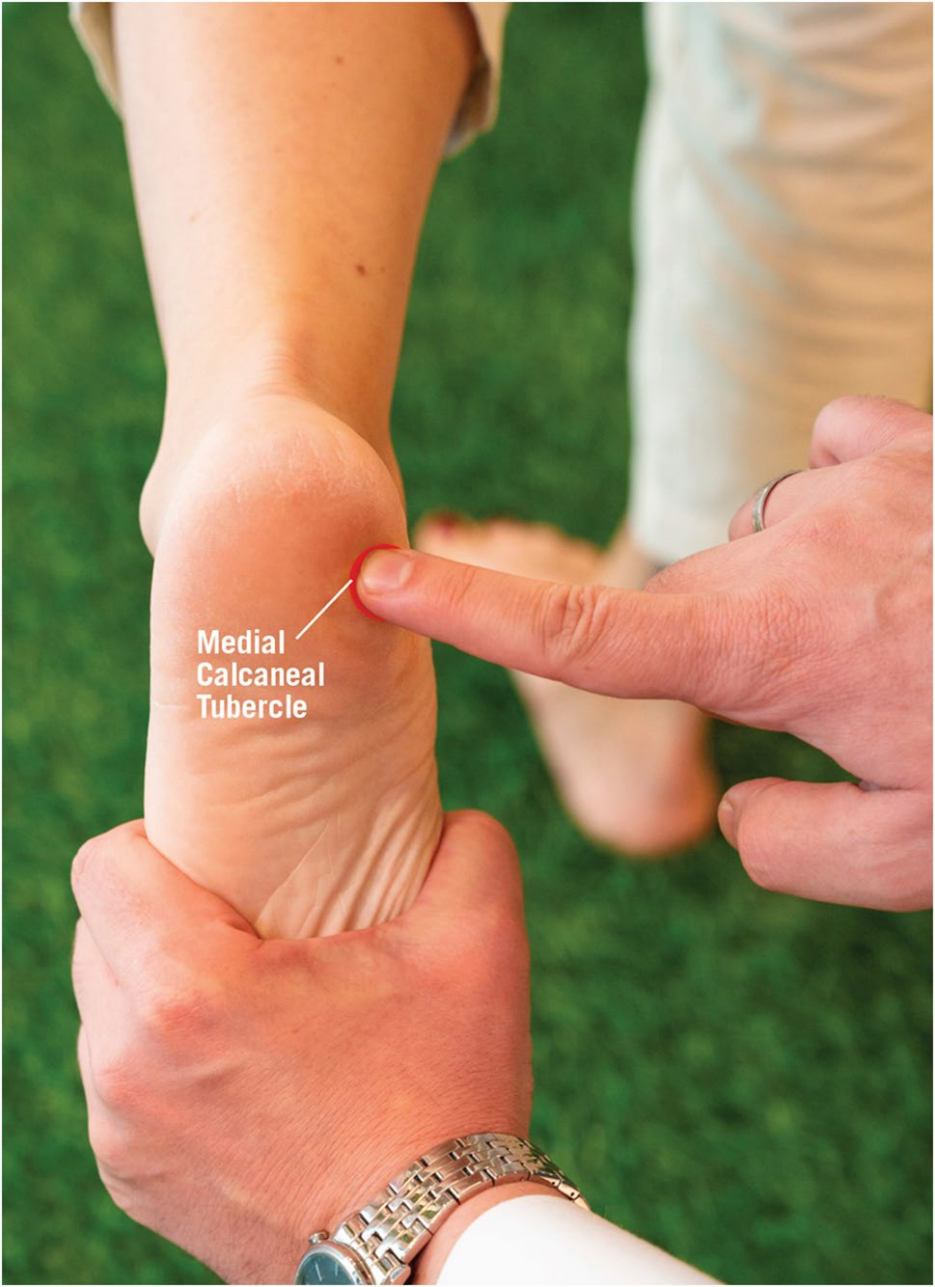
Medial calcaneal tuberosity tenderness: Tenderness and paresthesias may be identified, and the pain may radiate proximally and distally. Reprinted with permission of the Aspetar journal

The first branch of the lateral plantar nerve has sensory and motor fibers that innervate the calcaneal periosteum, long plantar ligament, lateral plantar skin, and intrinsic foot muscles, such as the abductor digiti minimi and flexor digitorum brevis. It usually originates near the tibial nerve bifurcation and can be prone to entrapment while navigating muscular and fascial layers, especially anterior to the calcaneal tuberosity. (Fig. 6).

ICN entrapment can result from muscle hypertrophy, bone spurs, and excessive rearfoot and midfoot pronation, compressing the nerve at certain points. Other causes include soft tissue growths, tendon inflammation, muscular changes, osseous abnormalities, trauma, and systemic issues like diabetes. [34].

## Clinical presentation and diagnosis

ICN entrapment is characterized by chronic pain localized to the plantar medial heel, with maximal tenderness over the nerve's course. Palpation of the plantar medial heel, particularly near the proximal abductor hallucis (AbH) or plantar fascia, often reproduces the characteristic pain. The hallmark finding of ICN entrapment is focal tenderness at the compression site, where the nerve is trapped between the AbH fascia and the quadratus plantae muscle. [1]. Patients may report tingling or electric sensations at the entrapment site. In advanced cases, weakness in the small toe's abduction may be seen.

The diagnostic value of electromyography (EMG) and nerve conduction velocity (NCV) studies is debated, as they may not reliably detect nerve dysfunction in ICN entrapment. [35]. Patients with medial calcaneal nerve involvement may show weakness in the AbH muscle. Symptoms worsen with pressure under the AbH when the foot is plantarflexed and inverted. Dorsiflexion increases tension on the plantar fascia, possibly worsening pain. Greater tenderness with relaxed plantar fascia suggests ICN entrapment as the cause. [10].

## Treatment approaches

Conservative treatment is key for managing ICN entrapment and relieving symptoms. Suggested methods include heel cups, pads, and heel lifts for pressure redistribution. A structured stretching program for the Achilles tendon and plantar fascia is helpful. NSAIDs provide pain relief, and local corticosteroid injections may temporarily relieve symptoms and assist in diagnosis.

If conservative measures fail to relieve symptoms, surgical decompression of the nerve may be an option. This typically involves releasing the fascia to relieve pressure on the nerve. It's crucial to set realistic expectations for surgical outcomes, as complete symptom resolution isn't always guaranteed. [36, 37].

## Conclusion

This review explores foot and ankle tunnel syndromes, which significantly impact patients and pose diagnostic challenges. These syndromes result from nerve compression in confined spaces, causing symptoms like sensory impairments and motor dysfunction. Understanding the causes, clinical signs, and treatment options is crucial for healthcare providers managing neurovascular symptoms. Differentiating these syndromes from other similar conditions requires a careful clinical evaluation, including patient history and diagnostic testing. Management should be tailored, starting with conservative approaches such as physical therapy, orthotic support, and medications. If these fail, surgery may be needed to relieve compression. Timely treatment is vital to prevent permanent nerve damage and improve outcomes.

## Data Availability

No datasets were generated or analysed during the current study.
